# Philadelphia Obstetrical Society

**Published:** 1889-05

**Authors:** J. M. Baldy

**Affiliations:** Secretary


### Society Reports.

### *PHILADELPHIA OBSTETRICAL SOCIETY.*

REPORTED BY J. M. BALDY, M. D., Secretary.

THURSDAY, March 7, 1889.

Dr. THEOPHILUS PARVIN in the chair.

Dr. J. M. BALDY read a paper on "Complications following Abdominal Section," which will be found on page 597.

DISCUSSION.

Dr. WM. GOODELL wholly agreed with the writer of the paper in regard to the stubborn nature of these fistulas, and to the impossibility of avoiding them. He had now three cases of fistula. One had followed the removal of an intraligamentary cyst, in which he had reopened the wound for bleeding. She recovered, but a fecal fistula had made its appearance about the fifth day, and had never closed. He had to peel off the tumor from the rectum. It was now a year and a half since the operation, and she was in the hands of a competent surgeon in the country. The only annoyance to the patient is an escaping gas from the wound. The second case followed the prolonged use of the drainage-tube after abdominal section for pelvic abscess. He had not had charge of the after-treatment, but she was in the hands of a skilful surgeon. It may be needful yet to make a counter-opening in the vagina. The third case was one of recurrent intraligamentary cyst. The fistula resulted from a previous operation in which the surgeon used the clamp many years ago. He operated last November, and removed a recurrent cyst lying in a very large abscess cavity. A drainage-tube was then used, which he still kept in, because he could not get the fistula to heal from the bottom. To-day he made an application of iodine to the track, and told the husband to repeat it daily for a time.

He has had cases of fistula where the drainage-tube was not used, but these were due to non-encapsulation of the pedicle ligatures. In one case, while using the syringe, the ligature was washed out. This gave him the cue, so, in the others, he fished the ligature out by means of a small hook on the end of a fine wire. On the other hand, in the case of an ovarian abscess, he had kept drainage up for several months, and yet the track closed. It is his intention, in a third case, to pass nitric acid to the bottom of the fistula and see what can be accomplished in this way. In this case she menstruates through the track. He thought if operators would wait some time after they have operated, before reporting their cases, they would find a number of hernias. He takes a good deal of pains to avoid this accident and close the abdominal wound in an analogous way to that practised by Dr. Price. The tendon, when retracted, he brings forward as much as possible with forceps, so as to bring it in contact with its fellow. He has had cases, in spite of every care. In the official report of Imlach's cases, although these were all cases of oöphorectomy, needing a very short incision, the percentage of hernias was fifteen. He keeps his patients in bed for two weeks before allowing them to sit up. In two cases in which he removed the ovaries in fibroid tumors, he has had the incision rupture from too early taking out of the stitches. In these cases, he sometimes leaves them in for two weeks. One case went home nineteen days after operation, in spite of orders, and the train becoming derailed, the jarring forced the cutaneous part of the wound open. Stitches had to be put in.

Dr. M. Price did not think, with Dr. Baldy, that abdominal surgery had anything to regret in these cases. He admitted that much of the dirt and filth, and many of the accidents which follow these terrible operations are actually due to the surgeon. He did not wonder at there being fistulous tracks, for the reason that in many of these cases the adhesions to the bowel are of such strength that their separation often removes everything down to the mucous coat. He has seen as much as six or seven sutures applied to such a case. Fistula is a repetition of the old abscess, which finds its way to the surface through the drainage track. All of the disease has not been removed. In many cases the fistula saves the woman's life and gives the surgeon a path through which to perfect his otherwise imperfect work. Fistula is a proof that the case has not been properly cared for. He did not believe that thirty per cent., or even five per cent., represented the number of hernias. He has only seen two cases follow. Their closure is unattended with danger. If due care and cleanliness are observed, fistulas will not occur.

Dr. J. Price was a little surprised that one so deeply interested as Dr. Baldy was in this subject of abdominal surgery, should stimulate vicious elements to criticism of our present position, especially since they now had their attention turned towards surgery of the brain, spinal cord, etc. Dr. Baldy speaks of hernia. The position of the incision, the condition of the abdominal walls, and the manner of introducing the sutures, are of great importance. Death has followed tight sutures, and he was satisfied that hernias often follow them. He always draws his sutures lightly. If you use three or four to the inch, tie them lightly, with perfect coaptation, your result will, as a rule, be perfect. In introducing his sutures, he takes in half as much skin as fascia, and twice as much fascia as peritoneum. This gives better apposition to the center of the wound. He has not had a suture-track abscess for more than a month, nor has there been any mischief about the tube. Nursing is of the greatest importance. The old nurses are meddlesome and dangerous, and he was glad to see them replaced by younger women. The tube can be dispensed with, very often, if the irrigation be thorough. Most surgeons are in too much of a hurry to get their patients up. Early rising is dangerous, and he has known surgeons to boast of getting their patients home in ten days. In fistulous tracks, through which menstruation occurs, the only thing to do is to tie the tube and release it from the abdominal incision. He has often had to deliver the bowel, in order to release adhesions, and even then has torn the bowel down to the mucous coat. This does no harm if the serous surfaces are brought together. A drainage-tube resting against these torn bowel surfaces favors the occurrence of a fistula. A man who gets scared at a fistula or ventral hernia is not prepared to do good work; his work begins in doubt and ends in disaster. The operation for curing a ventral hernia is not dangerous. We cannot ignore the importance of precision in diagnosis. We must try to decide as to the probable nature of the lesion. Dr. Baldy called attention to one point, that is the necessity of recognizing something definite on which to operate. Savage and others are satisfied to operate for subjective symptoms only. This is not right. The other day he refused to operate in a case which had multiple abscess in the lungs. Two weeks before, he had gone to the house prepared to operate, but the family had refused. The time will come when operators will be most arbitrary in these cases. We shall have the right to say, that if the general practitioner waits until the eleventh hour, we will not step in only when it takes a feather to depress the beam. Last Summer, a patient refused operation; to-day she sent for me and requested it. Peritonitis is often due to an imperfect toilet. It is quite often of limited extent, or localized, leaving adhesions to portions of the viscera. This is a common source of pain and discomfort. The only good remedy is to do the work over and release the adhesions. This past Summer, he had either done himself or assisted others to do eight of these operations over, and they had been the most difficult and trying of his whole experience. He wished to call attention to one case on which he had operated three times. Dr. Baldy saw the work. When he first saw her, pus was escaping from the umbilicus. He opened the abdomen, but failed to remove anything. Drainage was followed by a good recovery, but the wounds did not close. A year later he reoperated, but a fistulous track was left. Again, a year later, he used a catheter made of coils of wire. He passed this along around the ileo-pectineal line, towards the region of the kidney. He dissected along the pelvic bones, and irrigated through the catheter. Last week she was delivered of a fine baby, somewhere in the West. In this case he could find the ovaries, and there were no lesions of them or the tubes. This is the only case of pelvic abscess without tubal disease he had ever seen, in a long and rich experience. He wished to speak of two of the cases referred to by Dr. Baldy in his paper. One case he had operated on early in his experience, and had removed only one side of a specific tubal trouble. This he would never do again. The patient went into other hands and he did not care to refer to the ghastly surgery which followed. Another case of which he had personal knowledge, was a case of imperfect surgery. This was a large pus sac, which could have been removed but was drained. The woman died of psoas abscess. Skene says in his book, that pelvic abscess has frequently caused psoas abscess. The incomplete removal of diseased tubes should be rectified. If an inch of tube is left it will most likely do mischief. He has curretted into the cavity of the uterus, removing a cone-shaped piece. The tubes should be tied hard on to the uterus, and the ovaries should be tied at a good surgical neck, and the result will be about perfect.

Dr. Montgomery—We have become so enthusiastic in the field in which we are working as perhaps to overlook the dangers and difficulties that environ the way, and in our desire to defend and possibly to push forward our own work, we are sometimes led not to report our disasters. I think that Dr. Baldy has done us a kindness in dwelling on some of the disasters that may occur in abdominal operations. I am rather surprised to find that hernia is such a frequent lesion, in his experience. I have not found it so. The method of closing the wound, suggested by Dr. Price, is the one that I have largely used, and unless Dr. Baldy has come across some case which I do not know, I have never had a hernia in my experience. Fistulas with a constant discharge are exceedingly depressing and distressing. I have thought that drainage per vaginam might be preferable, where this accident is liable to occur. In such a case if a fistula did follow, it would not be so bad as if it were in the abdomen. I operated last Fall on a case in which half a gallon of broken-down blood was removed from a sac. The sac was drained, but death occurred in a few days. The post-mortem showed an abscess below the sac, which would have been opened if vaginal drainage had been made. The after-treatment is exceedingly important in many cases. These results are no doubt due to the fact that there still remains some diseased tissue about the ligaments or uterus. Where the tubal disease is gonorrheal, it is very hard to tie close enough to the uterus to remove all the pyogenic membrane. Even where we do, the inflammatory condition is still present in the uterus. The tendency of the extension of such inflammation to the pelvic tissues is in many cases the cause of after-trouble.

Dr. Hirst—In three cases he had lately to deal with, fistulæ directly followed laparatomy. One woman died a year after the operation, in consequence of this complication. In one case of great interest a mass of ligature was fished up, but the fistula still remains. After waiting some time he opened the vault of the vagina behind the uterus, on to the point of a sound passed into the fistula from above. He did not think he could have opened the bladder, but a vesical fistula must have already existed, for when he cut through the vault of the vagina, urine gushed out. A drainage-tube was put through the whole track, but now four months have passed and the woman is dying. He should hardly think the use of nitric acid free from danger, used as recommended by Dr. Goodell.

Dr. Hoffman—The paper of Dr. Baldy is iconoclastic. He looks at the matter from the wrong side. He collects a number of bad cases and puts them forward as an illustration of all abdominal surgery. If we look at his collection in the light of the fact that each case represents but a small proportion of the work of each man, the percentage of bad cases will be found to be almost infinitesimal. I myself do not believe that in the light of the bad showing which Dr. Baldy has made, if he to-morrow met with a case such as Dr. Price has referred to, he would hesitate one moment to operate. He would trust to doing his work well, and would feel sure that in nine cases out of ten he would have a good result. A report in a journal, a few days ago, shows that a prominent operator caused two deaths because he did not know how to tie the ligature. If a man does not know how to tie a ligature, that is no reason why abdominal surgery should be condemned. In my own experience I have never had a fistula follow these operations, nor have I had a hernia. Early rising is wrong. I know of an operator who boasted that his patient had gotten up at the end of a week, rode home, and walked up two flight of stairs to her room. I do not know whether she had a hernia or not, but if she did not she should have had one.

Dr. Baldy—I did not bring these cases forward as an objection to abdominal surgery, nor would they, nor many more, stay my hand if I found a case which required operation. My desire was to call direct attention to such accidents as these, and to stimulate our efforts to prevent their frequency. Nor is this by any means a complete list of all the cases on which I could put my hands. I could add dozens to the ones I have named. These cases have occurred in the hands of prominent men—men who profess to be teachers and who number their cases by the twenties, fifties, and hundreds. If we see such accidents in the hands of such men, we shall have more serious results in the hands of those less expert. Many cases of fistula can be avoided by care in the use of the drainage-tube. Few surgeons understand how to properly take care of a tube. I cannot agree with Dr. Price that fistulas always follow old fistula tracks, and are caused by diseased tissue left behind. In the majority of cases that I have seen the diseased tissue has all been removed, and the track occurs through what was formerly clean healthy tissue. I think that one common cause of hernia is the use of hemostatic forceps. These bruise the tissues, and if allowed to remain on too long cannot but irreparably damage the vitality of the parts included between the blades. The less we use the forceps the better union we will get. It is a rare occurrence that I have to use more than one or two pairs, sometimes three. These are always removed in a few moments, in fact as soon as I open the peritoneal cavity. They are no longer needed, and often if you are working through a small incision, are in the way. The fewer foreign bodies in and about the abdomen and abdominal wounds, the better for the patient and the easier for yourself. Cleanliness in all its details cannot be too strongly insisted on.

